# Transmission of Problem Gambling Between Adjacent Generations

**DOI:** 10.1007/s10899-020-09977-8

**Published:** 2020-09-22

**Authors:** David Forrest, Ian G. McHale

**Affiliations:** University of Liverpool Management School, Chatham Street, Liverpool, L69 7ZH UK

**Keywords:** Longitudinal data, Youth gambling, Problem gambling, Environmental influences

## Abstract

We investigate the extent to which gambling problems at age 20 are linked to parental gambling behaviour during childhood, employing data from a longitudinal study (ALSPAC) which has followed parents and children from Avon, England since pregnancy. 1058 children completed a problem gambling screen at age 20. When those children had been age 6, each of their parents was asked about their own gambling. We used regression to estimate the effect of parental gambling behaviour at child age 6 on the child’s problem gambling risk at age 20. Parental gambling participation at child age 6 was not a predictor of offspring problem gambling; but problem gambling by parents was a predictor of offspring problem gambling. However, this latter result was found only cross-gender (fathers’ behaviour influencing daughters and mothers’ behaviour influencing sons). This pattern was robust to models including measures of parental education and variables capturing family attitudes to health choices and the degree of domestic harmony. The sample illustrates high problem gambling prevalence amongst young adults. Although there is transmission of ‘problem gambling’ between generations, it appears to happen only cross-gender. This limits the importance of parental problem gambling as a source of the high prevalence because relatively few mothers exhibit problem gambling and risks to daughters from fathers are in the context of initially low baseline risks. Preventative policies might therefore be more appropriately targeted at young adults rather than rely on influencing parental gambling behaviour earlier in the child’s life.

**Electronic supplementary material:**

The online version of this article (10.1007/s10899-020-09977-8) contains supplementary material, which is available to authorized users.

## Introduction

As with many other physical and psychiatric illnesses, there may be an extent to which individuals are at greater risk of problem gambling where a parent has experienced the disorder. How common the transmission of parental gambling disorders to child gambling behaviour is will influence decisions on public health policy to mitigate prevalence and harm. Possible means of transmission include inheritance of genes and environmental influences during childhood. Studies of twins (for example, by Slutske et al. 2010) show that presence of problem gambling is more correlated between identical than between fraternal twins, controlling for exposure to similar environmental influences. Environmental influences are argued to include the effect of ‘modelling’ behaviour by parents whose heavy engagement ‘normalises’ gambling for their children and may indeed include introducing them to gambling, leading to elevated risk of problem gambling during adulthood (Valentine 2014).

Whatever the means of transmission, previous studies in various countries confirm that having a parent who experienced problem gambling is a risk factor for own problem gambling (Valentine 2014). However, these studies have weaknesses. First, they typically rely on respondents accurately reporting whether their parent was a problem gambler, raising the possibility of recall bias (Brewin et al. 1993) or bias from blaming parents for one’s own problems. Further, while they tend to report greater effects from paternal problem gambling than maternal problem gambling, it might in fact just be difficult to establish effects from mothers because prevalence of problem gambling will be low in this group. Third, as Valentine’s (2014) survey remarks, there is almost no literature investigating whether the risk for problem gambling is specifically related to problem gambling or alternatively to other issues correlated with parental problem gambling. For example, it is conceivable that parental problem gambling correlates with a range of family attitudes and lifestyle variables and it is these general household features rather than parental gambling behaviour itself which raise risk in the next generation.

In our study we were able to observe, for 1058 cases, information on a young person’s gambling activities and PGSI score at age 20 *and* information on parental gambling and problem gambling obtained from parents themselves when the young person was age 6. This allows us to test for association between problem gambling in young adulthood and parental gambling behaviour many years before. Additional data collected from each parent during the young person’s childhood allows us to test whether any association with parental gambling and problem gambling arises only because gambling behaviour serves as a proxy for general attitudes or dysfunctionality in the households where young adult problem gamblers were brought up.

## Data

Our data derive from the Avon Longitudinal Study of Parents and Children (ALSPAC), which has regularly gathered detailed information, mostly health-related, on a large sample of children and their parents born in Avon, UK in 1991–2. Details of the general sampling procedure from the inception of the Study are provided in online supplementary material. Ethical approval for the study was obtained from the ALSPAC Ethics and Law Committee. Informed consent for the use of data collected via questionnaires and clinics was obtained from participants following the recommendations of the ALSPAC Ethics and Law Committee at the time.

Each of the parents of the children in the sample analysed here had completed a questionnaire about his or her gambling behaviour, including the South Oaks Gambling Screen (SOGS), when the children were aged 6. Note that, for convenience, we will often use the word ‘fathers’ to refer to male ‘parents' although the male questionnaire was in fact completed by the mother’s partner at the time, who will not always have been the biological father. Of the mothers, 10.1% had been 21 years or under when the child was born; 62.3% had been aged 22–30; and the remaining 27.6% had been above 30.

The young people completed a questionnaire about their gambling behaviour, including the Problem Gambling Severity Index (PGSI) screen, at age 20. Therefore, we are able to examine the influence on outcomes in young adulthood of the behaviour of those who were responsible for the child at 6. But because those may not have included the biological father, it is impractical, and beyond our scope, to attempt to tease out biological and environmental influences on gambling outcome.

Of the young people for whom we had records from both parents’ gambling questionnaires at child age 6, 1058 took part in the new gambling module. 10.8% reported gambling weekly or more often. Those who had gambled at all in the previous 12 months took the PGSI problem gambling screen (derived from Ferris and Wynne 2001). The remainder were presumed not to be problem or at-risk gamblers. Of the 1058, 4.4% scored 3 or more on the PGSI scale, which classifies them as either at ‘moderate risk’ of experiencing gambling problems or unambiguously as ‘problem gamblers’. We group these sets of respondents together because there are too few observations in the ‘problem gambler’ band (PGSI ≥ 8) for meaningful analysis. The combined group we call ‘moderate problem or problem gamblers’ (MPPG). Among the males in our data set, the prevalence-rate of MPPG was 7.1% and among females 1.2%.

The parents had been surveyed when the children were age 6. They were asked about their participation in and level of engagement with gambling. They also completed a modified version of the SOGS screen for problem gambling (Lesieur and Blume 1987).^1^ To capture degree of engagement, we use number of regular gambling activities (where ‘regular’ means weekly-or-more participation and ‘activities’ refer to eleven named types of gambling, such as cards, horse bets, casino and bingo). In our modelling we also include each parent’s score on the reduced 12-item version of SOGS. 24.8% of fathers and 10.3% of mothers recorded a positive score, interpreted in this version of SOGS as signifying at least some level of problem with gambling. It may be noted that this is a much higher prevalence of problems than was reported above for the child cohort at age 20. This was to be expected because different screens were applied. The young people were assessed by the PGSI, which addresses problems experienced by the respondent in the preceding 12 months whereas parents completed a SOGS questionnaire which sought to identify lifetime prevalence, with questions in terms of ‘have you ever…?’. Further, the figures we quote refer to proportions of fathers and mothers who had a positive score on this version of SOGS, which is a low threshold. The proportions are consistent with responses for the same age-group according to data we accessed from the nearly contemporaneous British Gambling Prevalence Survey, 1999.

## Empirical Strategy

Throughout we estimated separate logistic regression models for the 20-year-old males and females, with MPPG status the variable to be explained. Separating by gender has the disadvantage that few cases of female MPPG (7) are observed. However, we were reluctant to merge male and female observations and represent the influence of gender only with a dummy variable, as is typical in the literature. Given the (invariably) very different problem gambling prevalence-rates between men and women, it seems implausible that the pattern of etiology of problem gambling is the same and therefore it is risky to force slope coefficient estimates in the regression models to be equal.

Our baseline models simply regressed MPPG on number of gambling activities of each parent and each parent’s problem gambling (SOGS) score. This identified simple correlations in the data.

Second, we introduced additional predictor variables describing the maximum level of education achieved by each parent. Based on information collected in mothers’ questionnaires at child age 12, binary variables indicated for mothers and fathers separately whether they had ‘A-levels or higher’, ‘GCSEs or equivalent’ (the reference category) or ‘no qualifications’. In the British system, A-levels are national academic examinations taken at about age 18; GCSE examinations are taken at about age 16. Level of qualifications may proxy socio-economic status and we wished to rule out that correlation of own problem gambling with parental problem gambling might just reflect socio-economic background rather than a direct influence of parental gambling activity and parental problem gambling.

Finally, we took our basic model and added variables, collected at various points in childhood, which reflected either the lifestyle of the family (in terms of risky and stigmatised behaviours such as smoking and frequent eating of fried food) or the degree of harmony in the family (proxied by self-reported frequency of parents arguing). Definitions of all these variables are presented in Table 1, together with the respective mean values. In the case of each binary variable, the mean is the proportion of observations falling within that category.

**Table 1:** Variable definitions and means

Variable	Definition	Mean
Mother's number of activities weekly	Number of (non-Lotto) gambling activities the mother reported participating in on a weekly basis, when the child was aged 6. Activities include: pools, bingo, slots, FOBTs, casino games, online betting, horse betting, dog betting, card games	0.41
Mother's problem gambling score	Number of positive responses the mother made to the modified version of the South Oaks Gambling Screen when the child was age 6	0.13
Partner's number of activities weekly	Number of (non-Lotto) gambling activities the mother’s partner reported participating in on a weekly basis, when the child was aged 6. Activities include: pools, bingo, slots, FOBTs, casino games, online betting, horse betting, dog betting, card games	0.58
Partner's problem gambling score	Number of positive responses the mother’s partner made to the modified version of the South Oaks Gambling Screen when the child was age 6	0.44
Mother has no qualifications	The mother reported no qualifications	0.02
Mother has above GCSEs	The mother reported her maximum education level was above GCSE (i.e. was either A-levels, degree, Masters, or PhD)	0.44
Mother's qualifications unknown	The mother’s qualifications were unknown	0.01
Partner has no qualifications	The partner reported no qualifications	0.04
Partner has above GCSEs	The partner reported his maximum education level was above GCSE (i.e. was either A-levels, degree, Masters or PhD)	0.43
Partner's qualifications unknown	The partner’s qualifications were unknown	0.02
Arguments at home	Equal to 1 if the mother reported arguing with her partner four or more times in the previous 3 months, when the child was age 12	0.28
Mother's BMI	The mother’s body mass index, calculated from height and weight as reported when the child was age 12	20.98
Partner's BMI	The partner’s body mass index, calculated from the partner’s height and weight as reported by the mother when the child was age 12	23.67
Fried food eaten more than twice weekly	Equal to 1 if the mother reports the household eats fried food at least once per week	0.10
Mother smokes	Equal to 1 if the mother reported smoking more than 0 times per week	0.107
Mother moderate or heavy drinker	Equal to 1 if the mother reports drinking more than 5 alcoholic drinks per week	0.486
Mother reports emotional problems	Equal to 1 if the mother reported having had emotional problems in the previous four weeks. Answered when the child was age 12	0.125
Mother lives with partner	Equal to 1 if the mother reported living with her partner when the child was age 12	0.977
Mother reports partner doesn’t always "loves child"	Equal to 1 if the mother reported feeling that her partner “loved” the child “sometimes” or “never”. Answered when the child was age 12	0.090
Partner smokes	Equal to 1 if the mother’s partner reported smoking more than 0 times per week	0.100
Partner active in last few weeks	Equal to 1 if the mother’s partner reported being able to participate in physical activity at a moderate or higher level of exertion	0.912
Partner in trouble with the law	Equal to 1 if the mother’s partner reported having ever been in trouble with the law	0.268

It is plausible that negative health behaviours and outcomes in adulthood may be linked to how much importance was attached to making healthy choices in the households where the subjects grew up. But if any one individual offspring health outcome is found to be correlated with a particular behaviour in the childhood home, the result may be spurious. Household health choices are correlated with each other and the parental behaviour observed may just be serving as a proxy for the attitudes behind their whole set of choices (Abrevaya and Tang 2011). Here, for example, risk of adult MPPG may be elevated where upbringing had been in a home tolerant of risky and stigmatised activities and it is this general attitude in the familial background, rather than parental gambling itself, which might explain the correlations in the data. Our ‘lifestyle variables’ reflect parental choices over a wide range of potentially healthy and unhealthy behaviours such that this set of variables should control for attitudes to risk in the parental home, allowing specific impacts from gambling to be identified.

A similar rationale accounts for our inclusion of a set of variables representing whether there had been a loving, caring environment or else conditions reflecting disharmony in the family home. Inter-spousal arguments or tensions associated with financial pressure on the household are common sources of such adverse developmental experiences. One recognised pathway to adult problem gambling is that of the ‘emotionally vulnerable problem gambler’ whose adverse developmental experience resulted in poor means of coping and a tendency to use gambling for escape or arousal (Blaszczynski and Nower 2002). Variables gathered from ALSPAC used here seek to capture negative and positive features of familial background. Again, the case for a specific influence on offspring MPPG from parental problem gambling would be strengthened if influences remained evident in the presence of a set of predictors capturing elements of dysfunctionality in the household where the child grew up.

## Results

Table 2 (Model 1) presents logistic regression results where only parental gambling variables collected at child age 6 are used to predict the risk of their child experiencing MPPG at age 20. Correlation between the number of regular gambling activities of a parent and his or her problem gambling score was positive (+ 0.22) but not sufficiently high to be problematic in terms of multicollinearity. Our ‘number of activities’ measure of gambling engagement was decisively non-significant for both father and mother in both male young person and female young person equations. However, strong effects are detected on to sons from maternal problem gambling and on to daughters from paternal problem gambling. Thus levels of parental engagement with gambling seems not to matter unless their gambling had shown signs of being problematic according to the SOGS screen. Where there was evidence that problem gambling was a risk to the following generation, transmission evident in the data was exclusively cross-gender (fathers to daughters, mothers to sons).

**Table 2:** Results of logistic regression models

Variable	Model 1		Model 2		Model 3	
	Males	Females	Males	Females	Males	Females
	Estimate (*t*-stat)	Estimate (*t*-stat)	Estimate (*t*-stat)	Estimate (*t*-stat)	Estimate (*t*-stat)	Estimate (*t*-stat)
Mother's number of activities weekly	0.111 (0.29)	− 0.430 (− 0.472)	0.020 (0.05)	− 0.149 (− 0.17)	0.084 (0.20)	0.220 (0.19)
Mother's problem gambling score	0.645 (2.56)	− 16.134 (− 0.01)	0.723 (2.70)	− 18.884 (− 0.01)	0.630 (2.07)	− 25.760 (− 0.01)
Partner's number of activities weekly	0.329 (0.99)	− 0.641 (− 0.96)	0.240 (0.70)	− 1.263 (− 1.71)	0.035 (0.09)	− 2.554 (− 1.83)
Partner's problem gambling score	0.079 (0.52)	0.697 (3.06)	0.061 (0.399)	0.912 (3.02)	0.141 (0.83)	1.627 (2.37)
Mother has no qualifications			− 0.549 (− 0.43)	− 17.628 (− 0.001)	− 0.354 (− 0.22)	− 16.250 (0.00)
Mother has above GCSEs			− 0.536 (− 1.18)	1.252 (1.53)	− 0.723 (− 1.52)	0.272 (0.19)
Mother's qualifications unknown			− 14.140 (− 0.01)	− 17.805 (− 0.001)	− 15.160 (− 0.01)	− 14.350 (0.00)
Partner has no qualifications			− 0.107 (− 0.11)	− 20.229 (− 0.002)	− 0.086 (− 0.08)	− 19.000 (0.00)
Partner has above GCSEs			− 0.153 (− 0.35)	− 19.505 (− 0.007)	− 0.193 (− 0.41)	− 20.270 (− 0.01)
Partner's qualifications unknown			0.727 (0.63)	− 18.387 (− 0.001)	0.592 (0.49)	− 16.730 (0.00)
Arguments at home					0.236 (0.56)	0.935 (0.75)
Mother's BMI					− 0.011 (− 0.22)	0.071 (0.54)
Mother smokes					0.026 (0.38)	− 0.207 (− 0.88)
Mother moderate or heavy drinker					− 0.454 (− 0.62)	1.874 (1.30)
Mother reports emotional problems					0.722 (1.22)	2.128 (1.38)
Mother lives with partner					0.463 (1.09)	1.237 (0.81)
Mother reports partner "loves child"					− 0.201 (− 0.31)	− 19.790 (0.00)
Partner smokes					13.890 (0.01)	14.480 (0.00)
Partner's BMI					0.804 (1.23)	− 0.594 (− 0.28)
Fried food eaten more than twice weekly					− 1.523 (− 1.37)	− 0.408 (− 0.29)
Partner active in last few weeks					− 0.923 (− 1.54)	− 3.677 (− 2.03)
Partner in trouble with the law					− 0.762 (− 1.32)	− 2.827 (− 1.25)
Constant	− 3.017 (− 9.74)	− 4.512 (− 8.60)	− 2.654 (− 6.81)	− 4.243 (− 5.32)	− 16.138 (− 0.01)	− 13.420 (0.00)
N	450	608	450	608	450	608
Log-likelihood	− 111.0	− 33.2	− 109.4	− 25.79	− 97.01	− 16.71
AIC	232.1	76.3	240.8	73.6	252.02	91.43

Effect sizes on MPPG from parental problem gambling scores were relatively high. Odds-ratios for mother-son and father-daughter effects were 1.91 and 2.01 respectively. For example, if a mother were among the 8.1% who scored 1 point on the modified 12-item SOGS screen, this was predicted to nearly double the odds of MPPG in the son relative to her being in the 89.7% of mothers who had a SOGS score of 0.

Our remaining empirical analysis checked the robustness of these findings to inclusion in the model of variables representing first parental education and then lifestyle and family harmony variables which may be correlated with problem gambling in the household. When we added dummy variables representing each parent’s education level (Table 2, Model 2), which may proxy socioeconomic status and cultural values, only one coefficient estimate was close to significance (mother has A-levels or higher, female equation, positive sign, *p* = 0.127). Coefficient estimates on parental problem gambling scores were similar as before in sign and significance. Therefore mother’s problem gambling remained an independent predictor of a son’s gambling problem at 20 and father’s problem gambling remained an independent predictor of daughter’s gambling problem at 20.

When we estimated equations including parental gambling variables and other variables describing the household at various points in the subject’s childhood (Table 2, Model 3), the additional variables failed to be jointly significant (*p* values of 0.19 and 0.18 in chi-square likelihood ratio tests). And few covariates added to represent household lifestyle and harmony were individually significant. There did appear to be a tendency for participation in physical activity self-reported by the father at child age 12 to be a protective indicator, and consumption of fried food by a mother at child age 12 to be a risk factor, for female MPPG at 20. However, the number of covariates (and therefore the number of hypothesis tests) was large, which makes it more likely that ‘significant’ results will appear purely by chance.

The pattern of results on the parental gambling variables themselves was similar to that in our baseline model though the size of the estimated effect of father’s SOGS score on the odds of the daughter experiencing MPPG was higher than in the baseline model (odds-ratio 4.54). Findings on the transmission of parental problem gambling to the following generation are therefore robust to the inclusion in the model of a set of variables which should together capture family background in terms of attitudes to health and domestic harmony.

## Discussion

The aim of the paper was to examine whether parental gambling behaviour during childhood affected the probability that a young adult would be experiencing gambling problems. We found that engagement by parents in gambling activities was not itself a predictor of problem gambling by their offspring. However, where fathers had had problems with their gambling, this was a risk factor for their daughters. Similarly, where mothers had had problems with their gambling, this was a risk factor for their sons. Only cross-gender transmission was evident in the data.

This finding of cross-gender transmission surprised us but is not without precedent for other negative behaviour patterns. Homel and Warren (2017) found that fathers’ binge drinking raised the risk of adolescent drinking by their daughters but not by their sons. Li et al. (2017) identified influence from mothers’ alcohol use disorder to early adult anti-social behaviour by sons (but not by daughters). To our knowledge, cross-gender transmission of problem gambling has not been noted in previous literature but it has not in fact been actively looked for since most research combines male and female observations in the receiving generation, potentially masking gender-specific transmission. Given that problem gambling is much more common among fathers than among mothers, this may have led to the conclusion by many authors, e.g. Vachon et al. 2004, that fathers are more important in raising risk for their offspring.

The strengths of the paper include that it is able to capture parental gambling behaviour through the self-report of parents themselves, recorded deep in the young adult’s childhood. Previous papers on young adult problem gambling have had to rely on subjects’ reports on whether there had been problem gambling in their family background. Further, we report separate models to account for young male and young female problem gambling whereas other literature has merged male and female observations. This has enabled us to identify cross-gender transmission. However, the study also has limitations. It studies only a group of children born in a particular English county in a particular year and, as we present a novel finding, it will depend on future research to reveal how generalizable that finding is. One particular problem of using the ALSPAC dataset is that, reflecting the make-up of Avon in the early 1990s, it has an overwhelmingly white-ethnic profile. It may therefore be unrepresentative of the population in Great Britain as a whole, which is more diverse and includes significant minorities among whom measured problem gambling prevalence is higher than for whites. However, it would be surprising if the result were not generalizable to white people in Great Britain, where we know from prevalence surveys, that the gambling behaviour of the relevant age group matches that observed here. Another limitation of our paper is that we observe parental problem gambling only once during a subject’s childhood. As gambling problems may be transient, some parents recorded as never having had gambling problems will in fact have been problem gamblers at some later stage during the offspring’s childhood. This would be expected to bias downwards our estimates of the impact of parental problem gambling. Additionally, it should be noted that parental gambling habits during adolescence might have different effects from those present in childhood. Another weakness is that there are few observations of MPPG in the data on young women. Findings from the model for young women should therefore be treated with caution.

While results identify transmission of problem gambling within families, the cross-gender routing limits the extent to which gambling problems among young adults can be linked to problem gambling among their parents. Many fewer mothers than fathers (10.3% compared with 24.8%) registered a positive score on the SOGS screen. There were therefore relatively few mothers to ‘pass on’ gambling problems to sons. Similarly, while paternal problem gambling appears to increase the odds that the daughter will have problems, this increased risk is applied to a baseline MPPG risk that is very low (compared with that for males aged 20). Thus a low absolute number of problem gambling cases would be expected to be generated from paternal problem gambling.

To some extent, results here validate public health messaging that parents should be mindful of the potential future effects of their own excessive gambling on their children when the latter reach adulthood. While we find no evidence that parental participation in gambling during childhood raises MPPG risk, we do find links between parental problematic gambling and children’s subsequent MPPG. On the other hand, transmission appears to work only cross-gender and transmission which occurs only cross-gender cannot account for the scale of MPPG observed in the data. MPPG is clearly much more prevalent among these twenty-year-olds than in the whole-population prevalence data (as reported in Conolly et al. 2018). To the extent that MPPG is so concentrated among those who have only recently reached the legal age for participation in most forms of commercial gambling, it may be argued that public health measures should target this vulnerable group by adopting new protective measures. For example, Licence Conditions in Great Britain oblige operators to intervene if customers exhibit patterns of play suggestive of harm. For those who are in the early years of exposure to legal gambling opportunities, thresholds for intervention could be required to be lower.

## Electronic supplementary material

Below is the link to the electronic supplementary material.

Supplementary file1 (DOCX 12 kb)

## Footnotes

1 ALSPAC used a reduced version of SOGS, with only twelve items. It included a question on whether the respondent had ever borrowed money for gambling but omitted follow-up questions on the source of borrowing (which can generate further points in the full SOGS screen).
