# Supplementary material for: Transmission of Problem Gambling Between Adjacent Generations
Source: J Gambl Stud. 2020 Sep 22;37(2):711–22. doi: 10.1007/s10899-020-09977-8 (PMC8144084; doi:10.1007/s10899-020-09977-8)
Supplement: Supplementary file 1 — Supplementary file1 (DOCX 12 kb) [file 10899_2020_9977_MOESM1_ESM.docx]

**Online supplementary material: The ALSPAC data set**

The Avon Longitudinal Study of Parents and Children (ALSPAC) was formerly known as ‘Children of the Nineties’ and continues till now to follow the lives of a sample of children born early in that decade in Avon County, England, with a focus on health-related issues. The Study website (<http://www.bristol.ac.uk/alspac/>) contains details of all the data that are available through a fully searchable data dictionary and variable search tool.

Here we describe the initial sampling strategy. ALSPAC recruited 14,541 pregnant women resident in Avon, UK with expected dates of delivery 1st April 1991 to 31st December 1992. 14,541 is the initial number of pregnancies for which the mother enrolled in the ALSPAC study and had either returned at least one questionnaire or attended a “Children in Focus” clinic by 19/07/99. Of these initial pregnancies, there was a total of 14,676 foetuses, resulting in 14,062 live births and 13,988 children who were alive at 1 year of age. ^1-2^

When the oldest children were approximately 7 years of age, an attempt was made to bolster the initial sample with eligible cases who had failed to join the study originally. However, in the present study, we include in our sample only those young people from whose mothers and partners gambling data were obtained at child age 6. All of the cases we consider are therefore drawn from the original sample.

**References**

1. Boyd A., Golding J. Macleod J, . Lawlor DA, Fraser A, Henderson J, Molloy L, Ness A, Ring S, Davey Smith G. Cohort Profile: The ‘Children of the 90s’; the index offspring of The Avon Longitudinal Study of Parents and Children (ALSPAC)’. *International Journal of Epidemiology* 2013; 42:111-127.

2. Fraser A, Macdonald-Wallis C, Tilling K, Boyd A, Golding J, Davey Smith G. Henderson J, Macleod J, Molloy L, Ness A, Ring, S, Nelson SM, Lawlor, DA. Cohort Profile: The Avon Longitudinal Study of Parents and Children: ALSPAC mothers cohort. *International Journal of Epidemiology* 2013;42:97- 110.
